# Residential exposure to fast-food restaurants and its association with diet quality, overweight and obesity in the Netherlands: a cross-sectional analysis in the EPIC-NL cohort

**DOI:** 10.1186/s12937-021-00713-5

**Published:** 2021-06-16

**Authors:** Marjolein C. Harbers, Joline W.J. Beulens, Jolanda MA Boer, Derek Karssenberg, Joreintje D. Mackenbach, Femke Rutters, Ilonca Vaartjes, WM Monique Verschuren, Yvonne T. van der Schouw

**Affiliations:** 1 Julius Center for Health Sciences and Primary Care, University Medical Center Utrecht, Utrecht University, Utrecht, the Netherlands; 2 Department of Epidemiology and Data Science, Amsterdam UMC, Vrije Universiteit, Amsterdam Public Health Research Institute, Amsterdam, the Netherlands; 3 National Institute for Public Health and the Environment (RIVM), Bilthoven, the Netherlands; 4 Department of Physical Geography, Faculty of Geosciences, Utrecht University, Utrecht, the Netherlands; 5 Upstream Team, www.upstreamteam.nl, Amsterdam UMC, Amsterdam, the Netherlands

**Keywords:** Food environment, Fast-food, Diet quality, Obesity, Neighborhood

## Abstract

**Background:**

Unhealthy food environments may contribute to unhealthy diets and risk of overweight and obesity through increased consumption of fast-food. Therefore, we aimed to study the association of relative exposure to fast-food restaurants (FFR) with overall diet quality and risk of overweight and obesity in a sample of older adults.

**Methods:**

We analyzed cross-sectional data of the EPIC-NL cohort (*n* = 8,231). Data on relative FFR exposure was obtained through linkage of home address in 2015 with a retail outlet database. We calculated relative exposure to FFR by dividing the densities of FFR in street-network buffers of 400, 1000, and 1500 m around the home of residence by the density of all food retailers in the corresponding buffer. We calculated scores on the Dutch Healthy Diet 2015 (DHD15) index using data from a validated food-frequency questionnaire. BMI was categorized into normal weight (BMI < 25), overweight (25 ≤ BMI < 30), and obesity (BMI ≥ 30). We used multivariable linear regression (DHD15-index) and multinomial logistic regression (weight status), using quartiles of relative FFR exposure as independent variable, adjusting for lifestyle and environmental characteristics.

**Results:**

Relative FFR exposure was not significantly associated with DHD15-index scores in the 400, 1000, and 1500 m buffers (β_Q4vsQ1_= -0.21 [95 %CI: -1.12; 0.70]; β_Q4vsQ1_= -0.12 [95 %CI: -1.10; 0.87]; β_Q4vsQ1_ = 0.37 [95 %CI: -0.67; 1.42], respectively). Relative FFR exposure was also not related to overweight in consecutive buffers (OR_Q4vsQ1_=1.10 [95 %CI: 0.97; 1.25]; OR_Q4vsQ1_=0.97 [95 %CI: 0.84; 1.11]; OR_Q4vsQ1_= 1.04 [95 %CI: 0.90–1.20]); estimates for obesity were similar to those of overweight.

**Conclusions:**

A high proportion of FFR around the home of residence was not associated with diet quality or overweight and obesity in this large Dutch cohort of older adults. We conclude that although the food environment may be a determinant of food choice, this may not directly translate into effects on diet quality and weight status. Methodological improvements are warranted to provide more conclusive evidence.

**Supplementary Information:**

The online version contains supplementary material available at 10.1186/s12937-021-00713-5.

## Introduction

An unhealthy diet and overweight are important modifiable risk factors for a wide range of chronic diseases. In 2017, globally 11 million deaths and 255 million disability adjusted life-years were attributable to poor diets [1]. The prevalence of unhealthy diets and overweight has risen substantially over the past decades. In 2014, more than half of the European population was overweight [2], and worldwide, a trend towards increased healthy food consumption between 1990 and 2010 was off-set by an even larger increase in unhealthy food consumption [3]. Causes for these trends are complex and multifactorial, and relate to factors on both the individual, socio-economic, and environmental level.

The community food environment has gained recognition as an important environmental determinant of diet quality and weight status and can be defined as the number, type and location of food outlets in a certain geographical area [4]. In recent decades, food environments have changed towards increased availability of food outlets offering relatively less healthy foods. For example, in the Netherlands, the number of out-of-home eating locations such as food delivery outlets, restaurants, and fast-food restaurants (FFR) increased by 120 %, 35 %, and 6 %, respectively between 2004 and 2018 while the number of local shops (e.g., greengrocer, butcher, bakery) decreased [5]. Similar changes in the food environment have also been observed in the UK [6] and US [7].

Out-of-home eating locations such as FFR generally serve foods which are energy-dense and nutrient-poor, and often serve these in large portions promoting overconsumption [8]. Previously, fast-food consumption has been linked to increased risk of overweight and obesity [9] and poor diet quality [10–12]. In the past decade, the idea has gained traction that exposure to FFR may influence diet quality and weight status through increased consumption of fast-food. Despite its plausibility, the evidence base remains inconclusive, with various studies across the UK, Denmark, the Netherlands and Australia, associating FFR exposure to poor dietary outcomes such as increased fast-food [13–15] or meat consumption [16] and higher risk of obesity [14, 16], while others report null associations for FFR exposure with diet [11, 17, 18] or weight status [19, 20].

These previous studies mainly investigated the association of FFR exposure with dietary outcomes or weight status in middle-aged populations, while less is known about these associations in older populations. In high-income countries, approximately 49 % of the total disease burden is attributable to disorders in people aged 60 years old and older, with diet-related disorders such as cardiovascular disease being the leading contributor [21]. Primary and secondary prevention strategies in older age groups relating to the control of modifiable risk-factors, such as diet, may not only contribute to lower mortality and morbidity, but may also improve quality of life and physical function and foster personal independence [22]. As approximately 1 out of 10 older adults consumes fast-food at least 1–2 times a week [23], it is worthwhile to investigate whether FFR exposure affects diet quality or weight status in this specific population.

Additionally, previous studies mainly investigated absolute measures using counts or densities [11, 13–15, 17, 18, 20, 24–26] rather than relative measures of FFR exposure, in which exposure is often defined as the proportion of FFR relative to other food outlets or restaurants [16, 19, 27, 28]. As such, relative measures of FFR capture the availability of other competing food outlets that may co-locate with FFR, and therefore reflect the opportunities for (un)healthy food choice. Consequently, a relative measure may better predict dietary outcomes as compared to absolute measures of FFR availability [29].

The objective of the current study is to study the association between relative exposure to FFR in relation to overall diet quality, overweight and obesity in a large population of older adults. We hypothesize that high relative FFR is associated with lower diet quality and higher risk of overweight or obesity, through providing relatively more opportunities for unhealthy food purchases.

## Methods

### Study population

EPIC-NL is the Dutch contribution to the European Prospective Investigation into Cancer and Nutrition (EPIC), and consists of the MORGEN and Prospect cohorts for which recruitment took place between 1993 and 1997. The design and rationale EPIC-NL have been described previously [30]. Briefly, the MORGEN cohort includes 22,654 men and women aged 20–69 years who were randomly sampled from three towns across the Netherlands (Maastricht, Doetinchem and Amsterdam). Prospect includes 17,357 women aged 49–70 from Utrecht and vicinity who participated in the national Dutch breast cancer screening program. The study was conducted in accordance with the Declaration of Helsinki and the institutional review boards of the University Medical Center Utrecht and Medical Ethics Committee of TNO Nutrition and Food Research approved the study. Written consent was obtained from all participants.

In 2015, participants who responded to a follow-up questionnaire on electromagnetic radiation in 2011, who were still alive and residing in the Netherlands in 2015, and who provided informed consent (*n* = 13,421 from Prospect and MORGEN Amsterdam and Maastricht only; the Doetinchem cohort was not invited) were invited to fill out a food frequency questionnaire (FFQ) for which the response rate was 62.7 % (*n* = 8,409). From this population, we excluded those with extreme energy intake defined as those within the lower and upper 0.5 % of the ratio between energy intake and basal metabolic rate (*n* = 84). We further excluded participants who did not return the general questionnaire at follow-up (*n* = 94). Consequently, the population for analysis comprised 8,231 participants.

### Exposure assessment – relative FFR exposure

Data on relative FFR exposure was obtained through linkage of the 6-digit postal code from home address in 2015 with a commercial retail database from Locatus. Locatus is a commercial company that collects data (e.g., location, type) on food outlets in the Netherlands based on systematic field audits, which are conducted regularly (e.g., once per year for busy shopping areas and once per two to three years for less busy shopping areas). Additionally, field audits are complemented by surveys and telephone interviews with retailers, assuring up-to-date data. A recent validation study showed good to excellent agreement between the Locatus data and research field audits [31]. Although the participants included in this study were originally recruited in Amsterdam, Utrecht and Maastricht (1993–1997), approximately 40 % of study participants included in the present study still lived in the recruitment areas, whereas the remaining study participants had moved elsewhere (Additional File 1).

From the Locatus database, we extracted data on food retailers operating in 2015. FFR were defined as traditional fast-food restaurants, grill-rooms and take-away outlets. Using PCRaster Python (pcraster.eu), we calculated a relative measure of FFR exposure in a 400, 1000 and 1500 street-network buffer (e.g., the distance someone can cover using the street-network) around the home of residence. This was done by dividing the densities of FFR by the density of all food retailers in the corresponding buffer.

### Outcome assessment – diet quality and BMI

In 2015, dietary intake was assessed with a validated semi-quantitative food-frequency questionnaire, the FFQ-NL 1.0 [32]. The FFQ assessed habitual consumption of 160 food items in the preceding year, through questions on consumption frequency and consumed amounts. Estimated food group intake was validated against an average of 2.7 telephone-based 24-hour recalls. Spearman correlation coefficients between estimates of the FFQ and estimates from the 24-hour recalls were 0.66 for fruits, 0.61 for bread and bread products, 0.38 for meat, 0.29 for vegetables, 0.27 for fish, 0.20 for nuts, seeds and snacks, and 0.13 for legumes. Average daily energy intake was estimated using the Dutch food composition table from 2011 [33].

In order to assess diet quality, we calculated scores on the Dutch Healthy Diet 2015 index (DHD15-index) [34]. Studying overall diet quality in contrast to isolated food groups or nutrients is nowadays preferred in nutritional epidemiology, as overall dietary patterns capture synergistic properties of individual foods and are more likely to affect health/weight status as compared to consumption of individual foods [35]. The DHD15-index reflects adherence to the Dutch dietary guidelines as issued by the Health Council in 2015. The index consists of fifteen food groups for which participants are allocated points based on absolute intakes of the respective food groups, resulting in a score ranging from 0 to 150 (Additional file 2). For the present analysis three out of the fifteen original food groups were excluded in the calculation of the DHD15-score. First, the coffee component was dropped from the score as the FFQ did not differentiate between types of coffee (filtered vs. unfiltered). Secondly, we excluded the sodium component from the DHD15-score, as sodium intake was not reliably captured with the FFQ. Third, we excluded the alcohol component from the score since we deemed it to be inappropriate in the context of our research question as alcohol is usually not sold in FFR. Moreover, we did not have data on type of cereal product (wholegrain vs. refined) except for bread. Therefore, the scoring of the wholegrain component was based on bread only, with an intake equal to or above 90 g receiving the maximum score of 10 points, and a proportionate decrease in points with decreased intake to the point where participants are assigned with 0 points when consuming no wholegrain bread. Taken together, the DHD15-score could range between 0 and 120 with higher scores indicating better adherence to the dietary guidelines and thus better diet quality.

Lastly, we used Body Mass Index (BMI) as a measure of overweight and obesity. Weight and height were self-reported in the follow-up questionnaire in 2015. BMI was calculated by dividing the weight by the height squared. Participants were categorized as normal weight (BMI < 25 kg/m2), overweight (25 ≤ BMI < 30), or obese (BMI ≥ 30).

### Covariate assessment

At baseline, participants completed a general questionnaire providing data on age, sex and educational level. Given the high proportion of older women in EPIC-NL, individual educational level may not be representative of women’s socioeconomic position when for example their partner is more highly educated. Therefore, we included the highest attained household educational level of the participant or the partner as a covariate in our analyses. Household educational level was categorized into low (primary education or intermediate vocational education), moderate (higher secondary education), and high (higher vocational education or university). At follow-up, participants provided information and smoking status, which was was categorized into never, former and current.

Data on neighborhood level socioeconomic position (SEP) in 2014 and 2016 – based on neighborhood income, educational level and job status – was obtained from the Netherlands Institute for Social Research and linked to the address information. This provided a continuous summary measure of neighborhood SEP for each participant based on their address, with higher scores indicating higher neighborhood SEP. The score ranges from approximately − 8 to 3. The summary measure of neighborhood level SEP in 2014 and 2016 was averaged to approximate neighborhood level SEP in 2015. Data on urbanicity of the neighborhood in 2015 was obtained from Statistics Netherlands, providing a categorical variable for each participant indicating very high level of urbanisation (≥ 2,500 addresses per km²), high level of urbanisation (1,500–2,500 addresses per km²), moderate level of urbanisation (1,000–1,500 addresses per km²), low level of urbanisation (500–1,000 addresses per km²), and very low level of urbanisation (< 500 addresses per km²). Data were linked to the EPIC-NL database through 4-digit postal codes and GWB-codes (Gemeente Wijk Buurt codes, or City Neighborhood Area codes) for the neighborhood level SEP and level of urbanisation, respectively.

### Statistical analyses

Baseline characteristics are displayed as means with standard deviations (SDs) for normally distributed variables, medians and interquartile range (IQR) for non-normally distributed variables, and frequencies and percentages for categorical variables, across quartiles of relative FFR exposure.

We performed multiple imputation on missing data (*n* = 422 for smoking; *n* = 193 for level of urbanisation; *n* = 92 for BMI; *n* = 22 for household educational level, *n* = 16 for neighborhood SEP), using age, sex, cohort, physical activity, smoking status, educational level, neighborhood SEP, level of urbanisation, DHD-15 score, and BMI as predictor variables and using 20 imputation sets.

Given the fact that recruitment took place across three cities in the Netherlands, we tested for a multilevel-structure by including a random intercept and random slope for recruitment area, with a variance component covariance pattern. The model without the random slope showed better model fit based on lowest AIC. The Wald statistic for the random intercept for recruitment area was non-significant (*p* = 0.32), indicating that accounting for clustering of participants was not necessary. We performed multivariable linear regression, with quartiles of relative FFR exposure as the independent variable and DHD15-scores as the dependent variable in order to allow for categorical comparisons between participants with varying relative FFR exposure. Models were adjusted for confounding variables based on previous literature. Model 1 was adjusted for age at follow-up, sex, and cohort. Model 2 was additionally adjusted for smoking, household educational level, and energy intake. Model 3 was additionally adjusted for neighborhood level SEP and level of urbanisation. Model 4 was additionally adjusted for the total count of food outlets in the corresponding buffer. We checked the assumptions of linearity and homoscedasticity by plotting the standardized residuals against standardized predicted values. The plots indicated that there was no evidence of non-linearity or heteroscedasticity. We checked the assumption of multicollinearity by examining the correlation coefficients among predictor variables, and the corresponding variance inflation factors and tolerance values, which showed no indication of multicollinearity.

We performed multinomial logistic regression for weight status (overweight and obesity vs. normal weight) as dependent variable, with the lowest quartile of relative FFR exposure as the reference category. Model structure was similar as to the linear regression analysis.

 We checked effect modification by household educational level, neighborhood SEP, and level of urbanisation in both the linear and multinomial logistic regression analysis by including an interaction-term between the continuous variable of relative FFR exposure and the potential effect-modifier in fully adjusted models. We considered a *p*-value of < 0.20 to be indicative of possible effect modification. In sensitivity analyses, we excluded participants who lived at their address for < 1 year and examined the associations in strata of age. For the analyses on weight status, we also conducted two additional sensitivity analyses: one using four instead of three BMI categories as outcome variable (adding an underweight category, defined as BMI < 18.5), and another excluding energy intake as confounder from the model since it might well be an intermediate in the relative FFR exposure – weight status pathway.

Statistical analyses were performed using IBM SPSS Statistics 24 (IBM Analytics, United States of America, New York). A *p*-value of < 0.05 was considered to be statistically significant.

## Results

### Baseline characteristics

The baseline characteristics of the study population across quartiles of relative FFR exposure in the 400 m buffer are presented in Table 1. Baseline characteristics across quartiles of FFR proportion for the 1000 and 1500 m buffer are presented in Additional files 3 and 4, respectively. The median proportion of FFR in the 400 m buffer ranged from 0 (IQR: 0–0) in the first quartile to 0.29 (IQR: 0.22–0.34) in the fourth quartile; ranges for the 1000 and 1500 m buffers were somewhat smaller. On average, participants were 70 years old (SD = 10) and 80 % was female. Moreover, 31.4 % had a low educational level, 6.7 % were current smokers, 36.9 % was overweight and 12.6 % of the population had obesity. The average score on the DHD15-index was 73 (SD = 17).

**Table 1:** Participant characteristics across quartiles of FFR proportion in the 400 m buffer^a^

	Q1	Q2	Q3	Q4
N, (%)	2871 (34.9)	1240 (15.1)	2063 (25.1)	2057 (25.0)
Median FFR proportion	0.00 (0.00–0.00)	0.07 (0.06–0.09)	0.14 (0.13–0.17)	0.29 (0.22–0.34)
Age, y	70 ± 10	70 ± 11	71 ± 10	70 ± 10
Sex, n (%)				
Male	539 (18.8)	239 (19.3)	437 (21.2)	404 (19.6)
Female	2332 (81.2)	1001 (80.7)	1626 (78.8)	1653 (80.4)
Household educational level, n (%)				
Low	901 (31.5)	408 (33.0)	653 (31.7)	622 (30.3)
Moderate	730 (25.5)	283 (22.9)	482 (23.4)	515 (25.1)
High	1229 (43.0)	547 (44.2)	923 (44.8)	916 (44.6)
Smoking, n (%)				
Current	192 (7.0)	86 (7.3)	145 (7.4)	125 (6.4)
Former	1290 (47.4)	534 (45.6)	983 (50.2)	961 (49.2)
Never	1242 (45.6)	552 (47.1)	831 (42.4)	868 (44.4)
BMI, kg/m2	25.5 ± 4.2	25.5 ± 4.4	25.5 ± 4.3	25.6 ± 4.1
Weight status				
Normal weight, n (%)	1434 (50.4)	612 (49.9)	1035 (50.8)	990 (48.7)
Overweight, n (%)	1049 (36.9)	447 (36.5)	759 (37.2)	780 (38.4)
Obesity, n (%)	360 (12.7)	167 (13.6)	244 (12.0)	262 (12.9)
Kcal/d	1,891 ± 634	1,901 ± 661	1,894 ± 627	1,888 ± 647
DHD-15 food groups, g/day				
Vegetables	117 (69–171)	124 (76–181)	118 (68–171)	119 (69–174)
Fruit	165 (77–234)	187 (89–241)	156 (70–237)	149 (71–235)
Wholegrain bread	71 (22–107)	70 (21–106)	70 (19–106)	70 (22–106)
Legumes	6 (0–16)	6 (0–17)	9 (0–17)	6 (0–16)
Nuts	6 (1–19)	7 (1–21)	6 (1–20)	7 (1–20)
Dairy	266 (141–402)	268 (149–399)	263 (145–406)	254 (136–392)
Fish	14 (7–29)	15 (7–36)	14 (5–29)	14 (7–36)
Tea	340 (121–510)	340 (146–510)	340 (121–510)	340 (97–680)
Butter and solid fats	0 (0–5)	0 (0–6)	0 (0–6)	0 (0–6)
Oils and diet margarines	12 (3–32)	10 (3–31)	11 (3–30)	11 (3–30)
Red meat	40 (19–74)	39 (19–74)	39 (17–76)	41 (18–77)
Processed meat	25 (10–45)	23 (8–41)	23 (8–42)	22 (9–41)
Sweetened beverages and fruit juices	75 (11–176)	58 (6–182)	71 (7–176)	63 (7–175)
Neighbourhood socioeconomic status^b^	0.4 (-0.3; 0.9)	0.3 (-0.6; 1.1)	0.4 (-0.6; 1.1)	0.4 (-0.8; 1.1)
Level of urbanisation^c^, n (%)				
Very low level of urbanisation	593 (21.0)	456 (39.2)	839 (41.3)	546 (27.1)
Low level or urbanisation	924 (32.7)	273 (23.5)	609 (30.0)	723 (35.8)
Moderate level of urbanisation	683 (24.2)	184 (15.8)	226 (11.1)	408 (20.2)
High level of urbanisation	260 (9.2)	187 (16.1)	211 (10.4)	196 (9.7)
Very high level or urbanisation	366 (13.0)	63 (5.4)	146 (7.2)	145 (7.2)

^a^Continuous variables are presented as means (standard deviation) or as medians (p25 – p75)

^b^Higher scores represent higher neighbourhood socioeconomic status

^c^Very low level of urbanisation ≤ 500 addresses/km^2^; low level of urbanisation = 500–1000 addresses/ km^2^; moderate level of urbanisation = 1000–1500 addresses/ km^2^; high level of urbanisation = 1500–2000 addresses/ km^2^; very high level or urbanisation ≥ 2000 addresses/ km^2^. The following variables had missing data: smoking status (*n* = 422); level of urbanisation (*n* = 193); BMI (*n* = 92); household educational level (*n* = 22); neighbourhood socioeconomic status (*n* = 16)

### Association between relative FFR exposure and diet quality

The regression coefficients from the multiple linear regression models for the association between quartiles of FFR proportion and scores on the DHD15-index are presented in Table 2. Relative FFR exposure was not significantly associated with DHD15-index scores when comparing extreme quartiles in the fully adjusted model in the 400 m buffer (β = -0.21, 95 % CI: -1.12; 0.70), 1000 m buffer (β = -0.12, 95 % CI: -1.10; 0.87), or 1500 m buffer (β = 0.37, 95 % CI: -0.67; 1.42). Relative FFR exposure was associated with DHD15-index scores when comparing quartile 2 with quartile 1 in model 1 (β = 1.56, 95 % CI: 0.46; 2.66), model 2 (β = 1.46, 95 % CI: 0.41; 2.52), and model 3 (β = 1.37, 95 % CI: 0.29; 2.45). However, this association attenuated to non-significance after adjustment for the total presence of food retailers in model 4 (β = 1.10, 95 % CI: -0.07; 2.28). Excluding participants who had lived at their current address for < 1 year did not substantially alter findings (data not shown), and associations were similar in strata of age (data not shown). Furthermore, there was no significant effect modification by household educational level, neighborhood SEP, or level of urbanisation (all *p*-values > 0.20).

**Table 2:** Regression coefficients (95 %CI) for the association between quartiles of FFR proportion and DHD15-index scores

	Q1	Q2	Q3	Q4
	β (95 % CI)	β (95 % CI)	β (95 % CI)	β (95 % CI)
400 m buffer				
N, (%)	2871 (34.9)	1240 (15.1)	2063 (25.1)	2057 (25.0)
Median FFR (IQR)	0.00 (0.00–0.00)	0.07 (0.06–0.09)	0.14 (0.13–0.17)	0.29 (0.22–0.34)
Model 1^a^	ref	1.56 (0.46; 2.66)^e^	0.43 (-0.51; 1.36)	0.10 (-0.83; 1.04)
Model 2^b^	ref	1.46 (0.41; 2.52)^e^	0.28 (-0.62; 1.18)	-0.05 (-0.94; 0.85)
Model 3^c^	ref	1.37 (0.29; 2.45)^e^	0.05 (-0.87; 0.96)	-0.15 (-1.05; 0.75)
Model 4^d^	ref	1.10 (-0.07; 2.28)	-0.22 (-1.24; 0.81)	-0.21 (-1.12; 0.70)
1000 m buffer				
N, (%)	2057 (25.0)	2057 (25.0)	2044 (24.8)	2073 (25.2)
Median FFR (IQR)	0.00 (0.00–0.09)	0.13 (0.11–0.13)	0.16 (0.15–0.17)	0.21 (0.20–0.25)
Model 1^a^	ref	-0.22 (-1.22; 0.79)	0.38 (-0.63; 1.39)	-0.08 (-1.09; 0.93)
Model 2^b^	ref	-0.21 (-1.18; 0.76)	0.10 (-0.87; 1.07)	-0.15 (-1.12; 0.82)
Model 3^c^	ref	-0.48 (-1.45; 0.49)	-0.27 (-1.25; 0.71)	-0.34 (-1.31; 0.63)
Model 4^d^	ref	-0.82 (-1.82; 0.18)	-0.16 (-1.14; 0.82)	-0.12 (-1.10; 0.87)
1500 m buffer				
N, (%)	2057 (25.0)	2060 (25.0)	2128 (25.9)	1986 (24.1)
Median FFR (IQR)	0.10 (0.00–0.10)	0.13 (0.12–0.13)	0.17 (0.16–0.19)	0.22 (0.20–0.24)
Model 1^a^	ref	-0.38 (-1.39; 0.63)	-0.56 (-1.55; 0.44)	0.16 (-0.85; 1.18)
Model 2^b^	ref	-0.50 (-1.47; 0.47)	-0.53 (-1.49; 0.43)	-0.07 (-1.05; 0.91)
Model 3^c^	ref	-0.70 (-1.68; 0.27)	-0.66 (-1.63; 0.30)	-0.18 (-1.16; 0.81)
Model 4^d^	ref	-0.75 (-1.72; 0.23)	-0.27 (-1.27; 0.73)	0.37 (-0.67; 1.42)

^a^Model 1 is adjusted for age, sex, and cohort

^b^Model 2 is additionally adjusted for smoking, energy intake, and household educational level

^c^Model 3 is additionally adjusted for neighbourhood level socioeconomic status and level of urbanisation

^d^Model 4 is additionally adjusted for the total presence of food retailers in the corresponding buffer

^e^Indicates statistical significance

### Association between relative FFR exposure, overweight and obesity

The odds ratio’s from the multinomial logistic regression for the association between quartiles of FFR proportion and overweight and obesity are presented in Table 3. Relative FFR exposure was not significantly associated with overweight in the 400 m buffer (1.10; 95 % CI: 0.97; 1.25), 1000 m buffer (OR = 0.97; 95 % CI: 0.84; 1.11) and 1500 m (OR = 1.04; 95 % CI: 0.90; 1.20) in fully adjusted models when comparing extreme quartiles. Similarly, relative FFR exposure was not significantly associated with obesity in the 400 m buffer (OR = 1.08, 95 % CI: 0.90; 1.30), 1000 m buffer (OR = 0.98, 95 % CI: 0.81; 1.20), or 1500 m buffer (OR = 1.06, 95 % CI: 0.85; 1.31). Excluding participants who had lived at their current address for < 1 year did not substantially alter findings (data not shown), and associations were similar in strata of age (data not shown). There was no significant effect modification by educational level, neighborhood SEP, or level of urbanisation (all *p*-values > 0.20). Using four categories of weight status as outcome variable and excluding energy intake as confounder from the model did not materially alter the results (data not shown).

**Table 3:** Odds ratios (95 %CI) for the association between quartiles of FFR proportion and risk of overweight and obesity

	400 m buffer		1000 m buffer		1400 m buffer	
	Odds of being overweight	Odds of being obese	Odds of being overweight	Odds of being obese	Odds of being overweight	Odds of being obese
Model 1^a^						
Q1	1.00 (reference)	1.00 (reference)	1.00 (reference)	1.00 (reference)	1.00 (reference)	1.00 (reference)
Q2	0.99 (0.86; 1.15)	1.08 (0.88; 1.33)	0.89 (0.78; 1.02)	0.88 (0.73; 1.07)	1.08 (0.95; 1.24)	1.04 (0.86; 1.27)
Q3	0.97 (0.86; 1.10)	0.92 (0.77; 1.11)	0.91 (0.80; 1.04)	0.86 (0.71; 1.04)	1.06 (0.92; 1.21)	1.04 (0.86; 1.26)
Q4	1.07 (0.95; 1.21)	1.05 (0.88; 1.26)	0.97 (0.85; 1.11)	0.99 (0.82; 1.20)	1.07 (0.93; 1.22)	1.06 (0.87; 1.29)
Model 2^b^						
Q1	1.00 (reference)	1.00 (reference)	1.00 (reference)	1.00 (reference)	1.00 (reference)	1.00 (reference)
Q2	1.00 (0.86; 1.17)	1.10 (0.89; 1.36)	0.89 (0.77; 1.02)	0.88 (0.72; 1.07)	1.08 (0.94; 1.24)	1.03 (0.85; 1.26)
Q3	0.99 (0.87; 1.12)	0.94 (0.78; 1.13)	0.92 (0.81; 1.06)	0.88 (0.72; 1.07)	1.05 (0.91; 1.20)	1.03 (0.85; 1.25)
Q4	1.09 (0.96; 1.23)	1.07 (0.89; 1.29)	0.96 (0.84; 1.10)	0.97 (0.80; 1.18)	1.06 (0.93; 1.22)	1.05 (0.86; 1.29)
Model 3^c^						
Q1	1.00 (reference)	1.00 (reference)	1.00 (reference)	1.00 (reference)	1.00 (reference)	1.00 (reference)
Q2	1.01 (0.87; 1.17)	1.12 (0.90; 1.39)	0.90 (0.78; 1.03)	0.89 (0.73; 1.09)	1.08 (0.94; 1.24)	1.02 (0.84; 1.25)
Q3	1.00 (0.88; 1.13)	0.96 (0.80; 1.16)	0.95 (0.83; 1.09)	0.91 (0.74; 1.11)	1.04 (0.91; 1.20)	1.01 (0.83; 1.23)
Q4	1.09 (0.96; 1.23)	1.06 (0.88; 1.27)	0.98 (0.86; 1.12)	0.99 (0.81; 1.20)	1.08 (0.94; 1.24)	1.07 (0.87; 1.30)
Model 4^d^						
Q1	1.00 (reference)	1.00 (reference)	1.00 (reference)	1.00 (reference)	1.00 (reference)	1.00 (reference)
Q2	1.08 (0.91; 1.27)	1.19 (0.94; 1.51)	0.92 (0.79; 1.06)	0.89 (0.73; 1.10)	1.08 (0.94; 1.24)	1.02 (0.84; 1.25)
Q3	1.06 (0.92; 1.23)	1.02 (0.83; 1.26)	0.95 (0.83; 1.09)	0.91 (0.74; 1.11)	1.02 (0.88; 1.17)	1.00 (0.82; 1.22)
Q4	1.10 (0.97; 1.25)	1.08 (0.90; 1.30)	0.97 (0.84; 1.11)	0.98 (0.81; 1.20)	1.04 (0.90; 1.20)	1.06 (0.85; 1.31)

^a^Model 1 is adjusted for age, sex, and cohort

^b^Model 2 is additionally adjusted for smoking, energy intake, and household educational level

^c^Model 3 is additionally adjusted for neighbourhood level socioeconomic status and level of urbanisation

^d^Model 4 is additionally adjusted for the total presence of food retailers in the corresponding buffer

## Discussion

In the present study, we aimed to study the association of relative FFR exposure with diet quality, overweight and obesity. We did not confirm our hypothesis that a relatively unhealthy residential food environment – characterized by a high proportion of FFR relative to all food outlets – was associated with lower diet quality and higher risk of overweight or obesity.

 Although the evidence base on food environment research is large, substantial heterogeneity exists in the way FFR exposure (e.g., relative vs. absolute measures) and diet outcomes (e.g., fast-food purchasing, fast-food consumption, diet quality) are operationalized. The only other study that examined relative FFR exposure with a diet quality score was conducted in a sample of younger adults from the US. It found that relative FFR exposure expressed as the ratio of FFR to total restaurants was over time associated with lower scores on a diet quality index using instrumental variable analysis, but was not in ordinary least squares regression models [28]. This suggests that residual confounding attenuates associations between relative FFR exposure and diet quality in non-causal models. Since our results were based on ordinary regression models, this may explain the null findings observed in the present study. Three other studies investigated the association of absolute FFR exposure with diet quality scores, showing null findings as well [11, 17, 18]. However, comparison of studies using different measures of FFR exposure (e.g., relative vs. absolute) should be done with caution, as these measures represent different aspects of the FFR environment [36].

As for associations of relative FFR exposure with weight status, evidence seems somewhat more conclusive. Previously, relative FFR exposure expressed as the ratio of FFR to all food outlets has been associated with higher BMI and higher odds of obesity (in two out of four buffers studied) in Australia [19] and the UK [16], respectively. Similarly, relative FFR exposure expressed as the ratio of FFR to total restaurants was associated with higher BMI and increased odds of obesity in Canada [27]. These findings contrast with the null findings observed in the present study, and may be attributed to the relatively high age of our study population (on average ~ 70 years). Indeed, there is evidence that suggests that consumption of take-away meals declines with age [23], which may be due to the fact that older adults have less income [37], experience reduced mobility [26] (e.g., go outside less, so are less susceptible to the tempting food environment) or did not acquire the habit of obtaining fast-food in their youth as fast-food restaurants were less proliferate [38]. Moreover, other methodological differences (e.g., different model adjustment and buffer sizes) or different geographical contexts could account for the contrasting findings.

Besides abovementioned explanations for the contrasting findings of the present study in light of previous evidence, we cannot not preclude the possibility that null associations with diet quality and weight status are true null findings. For example, a recent study by Hobbs et al. challenged the hypothesis that FFR exposure is associated with diet quality and BMI, and alternatively suggested that area-level deprivation rather than FFR exposure may account for associations that have previously been observed with BMI and diet quality through residual confounding [17]. Indeed, using a structural equation modelling approach, Hobbs et al. showed that area-level deprivation was associated with higher absolute FFR exposure, higher BMI and lower diet quality, whereas FFR exposure was not associated with diet quality of BMI. Although this study used absolute FFR exposure rather than relative FFR exposure, it may explain why we were unable to confirm our initial hypothesis.

The present study should be interpreted in light of several limitations. First, this study employed a cross-sectional design, which does not allow to draw conclusions on the temporality of the association. Moreover, weight and height were self-reported which may have resulted in non-differential outcome misclassification and bias towards the null. Moreover, we only measured exposure to FFR around the home, which is likely to provide an underestimation of an individual’s true exposure to FFR [14]. However, given the older population in the present study, we might expect that activity patterns will generally be centered around the home to a greater extent as compared to younger populations [39]. Also, it has been hypothesized that individuals may differ in the extent to which they are susceptible to the food environment [18, 25]. However, we lacked data on behavioral constructs that may moderate the association between the food environment, diet and weight status. Additionally, participants in cohort studies generally represent a more health-conscious population. Given the fact that we only included participants that took part in the data-collection at follow-up (18–22 years after inclusion in the cohort), we cannot exclude the potential of healthy volunteer bias. As a result, our study population may have been generally health-conscious and not likely to obtain FFR, leading to our null associations. Last, although we adjusted for a wide range of potential confounders, we cannot exclude possibility of residual confounding. For example, we used educational level as a proxy of SEP and did not have data on income, which may be an important confounder as well.

Strengths of the present study include the use of a validated FFQ which allowed us to compute a comprehensive measure of diet quality, opposed to short screeners used often in food environment research [40]. Lastly, the study population included participants from various areas in the Netherlands, providing a geographically diverse sample and assuring variation in relative FFR exposure.

## Conclusions

In the present study among predominantly older women we did not find any evidence of an association of relative FFR exposure around the home with diet quality or weight status. Although the food environment may be a determinant of food choice, this may not directly translate into effects on diet quality and weight status. Methodological improvements in accurately assessing FFR exposure and outcomes, and incorporating behavioural moderators, are warranted to provide more conclusive evidence.

## Supplementary Information

**Additional file 1.** Distribution of home locations in 2015 in the study population.

**Additional file 2.** Components of the DHD15-index and corresponding dietary recommendations and their threshold (minimum score) and cut-off (maximum score) values.

**Additional file 3.** Participant characteristics across quartiles of FFR proportion in the 1000m buffer.

**Additional file 4.** Participant characteristics across quartiles of FFR proportion in the 1500m buffer.

## Data Availability

The datasets used and/or analysed during the current study are available from the Principal Investigator of EPIC-NL (YTvdS) on reasonable request.
