# Supplementary figures and images for: Residential exposure to fast-food restaurants and its association with diet quality, overweight and obesity in the Netherlands: a cross-sectional analysis in the EPIC-NL cohort
Source: Nutr J. 2021 Jun 16;20:56. doi: 10.1186/s12937-021-00713-5 (PMC8210363; doi:10.1186/s12937-021-00713-5)

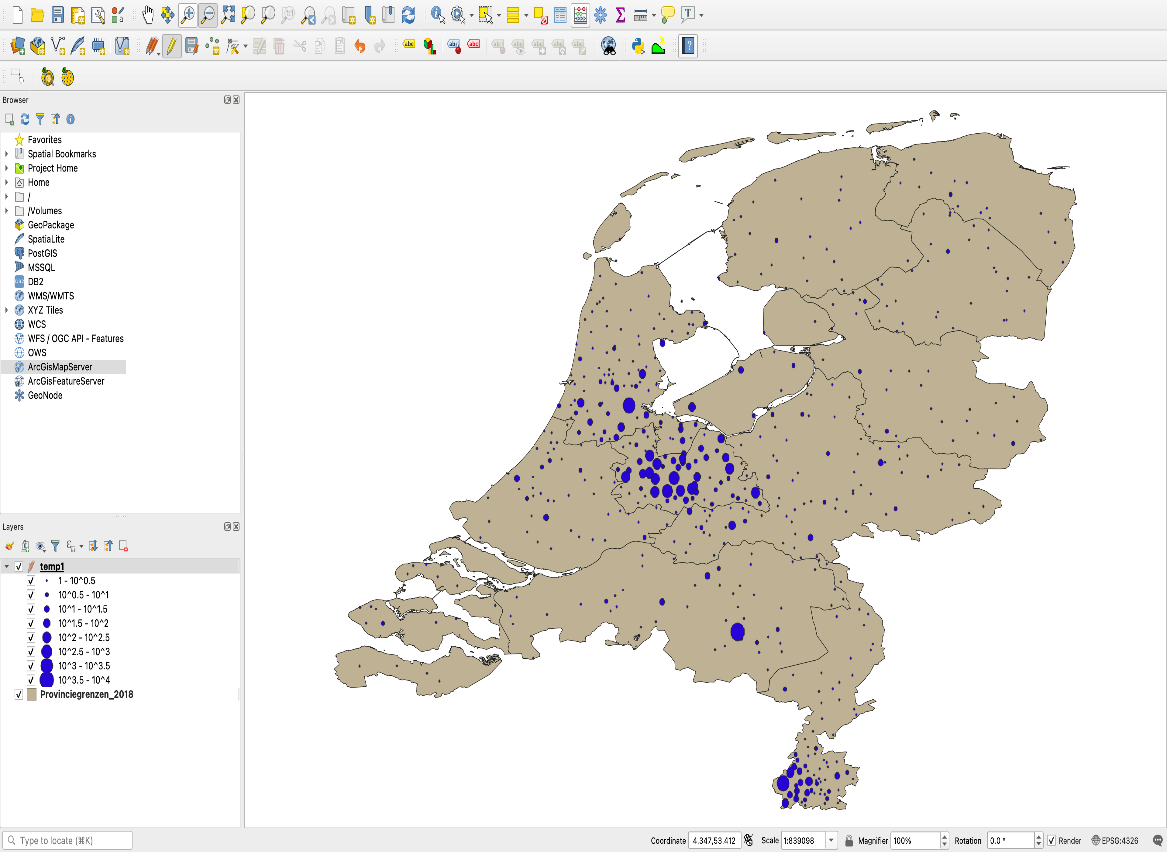


**Additional file 1.** Distribution of home locations in 2015 in the study population.

Supplement: Supplementary file 1 — Additional file 1. Distribution of home locations in 2015 in the study population. [file 12937_2021_713_MOESM1_ESM.docx]
