# Supplementary material for: Residential exposure to fast-food restaurants and its association with diet quality, overweight and obesity in the Netherlands: a cross-sectional analysis in the EPIC-NL cohort
Source: Nutr J. 2021 Jun 16;20:56. doi: 10.1186/s12937-021-00713-5 (PMC8210363; doi:10.1186/s12937-021-00713-5)
Supplement: Supplementary file 2 — Additional file 2. Components of the DHD15-index and corresponding dietary recommendations and their threshold (minimum score) and cut-off (maximum score) values. [file 12937_2021_713_MOESM2_ESM.docx]

**Additional file 2.** Components of the DHD15-index and corresponding dietary recommendations and their threshold (minimum score) and cut-off (maximum score) values.

| DHD15-component | Dietary recommendation | Minimum score (0 points) | Maximum score (10 points) |
| --- | --- | --- | --- |
| 1. Vegetables | Eat at least 200 g of vegetables daily | 0 g/d | ≥ 200 g/d |
| 1. Fruit | Eat at least 200 g of fruit daily | 0 g/d | ≥ 200 g/d |
| 1. Whole grain products^a^ | a. Eat at least 90 g of wholegrain products daily | 0 g/d | ≥ 90 g/d |
|  | b. Replace refined cereal products by whole-grain  products | No consumption of whole grain products OR ratio whole grain to refined grain ≤ 0.7 | No consumption of refined products OR ratio of whole grains to refined grains ≥ 11 |
| 1. Legumes | Eat legumes weekly. | 0 g/d | ≥10 g/d |
| 1. Nuts | Eat at least 15 grams of unsalted nuts a day. | 0 g/d | ≥15 g/d |
| 1. Dairy^b^ | Eat a few portions of dairy produce daily, including  milk or yoghurt. | 0 g/d OR ≥750 g/d | 300–450 g/d |
| 1. Fish^c^ | Eat one serving of fish weekly, preferably oily fish. | 0 g/d | ≥15 g/d |
| 1. Tea | Drink three cups of black or green tea a day | 0 g/d | ≥450 g/d |
| 1. Fats and oils | Replace butter, hard margarines, and cooking fats by  soft margarines, liquid cooking fats, and vegetable oils | No consumption of soft margarines, liquid cooking fats and vegetable oils OR ratio of liquid cooking fats to solid cooking fats ≤ 0.6 | No consumption of butter, hard margarines and cooking fats OR ratio of liquid cooking fats to solid cooking fats ≥ 13 |
| 1. Coffee^d^ | Replace unfiltered coffee by filtered coffee. | Any consumption of unfiltered coffee | Consumption of only filtered coffee OR no coffee consumption |
| 1. Red meat | Limit consumption of red meat. | ≥ 100 g/d | ≤45 g/d |
| 1. Processed meat | Limit consumption of processed meat. | ≥ 50 g/d | 0 g/d |
| 1. Sugar-sweetened beverages and fruit juices | Limit consumption of sweetened beverages and fruit  juices. | ≥ 250 g/d | 0 g/d |
| 1. Alcohol | If alcohol is consumed at all, intake should be limited  to one Dutch unit (10 gram ethanol) daily | Women: ≥20 g ethanol/d  Men: ≥30 g ethanol/d | Women: ≤10 g ethanol/d  Men: ≤10 g ethanol/d |
| 1. Sodium^e^ | Limit consumption of table salt to 6 g daily | ≥ 3.8 g sodium/d | ≤ 1.9 g sodium/d |

^a^The wholegrain component comprises two sub-components of which each sub-component has a maximum score of 5 points; ^b^For the dairy component, a maximum of 40 g cheese can be included. ^c^For the fish component, a maximum of 4 g lean fish can be included. ^d^The coffee component was not included in the calculation of the DHD15-score as no data was available on type of coffee (filtered/unfiltered). ^e^Sodium only originated from foods; intake from added salt was not captured by the FFQ.
