# Supplementary material for: Residential exposure to fast-food restaurants and its association with diet quality, overweight and obesity in the Netherlands: a cross-sectional analysis in the EPIC-NL cohort
Source: Nutr J. 2021 Jun 16;20:56. doi: 10.1186/s12937-021-00713-5 (PMC8210363; doi:10.1186/s12937-021-00713-5)
Supplement: Supplementary file 3 — Additional file 3. Participant characteristics across quartiles of FFR proportion in the 1000m buffer. [file 12937_2021_713_MOESM3_ESM.docx]

**Additional file 3.** Participant characteristics across quartiles of FFR proportion in the 1000m buffer^a^.

|  | Q1 | Q2 | Q3 | Q4 |
| --- | --- | --- | --- | --- |
| N, (%) | 2057 (25.0) | 2057 (25.0) | 2044 (24.8) | 2073 (25.2) |
| Median FFR proportion | 0.00 (0.00 - 0.09) | 0.13 (0.11 - 0.13) | 0.16 (0.15 - 0.17) | 0.21 (0.20 - 0.25) |
| Age, y | 70 ± 10 | 70 ± 10 | 69 ± 10 | 70 ± 10 |
| Sex, n (%) |  |  |  |  |
| Male | 398 (19.3) | 392 (19.1) | 430 (21.0) | 399 (19.2) |
| Female | 1659 (80.7) | 1665 (80.9) | 1614 (79.0) | 1674 (80.8) |
| Household educational level, n (%) |  |  |  |  |
| Low | 654 (31.9) | 671 (32.7) | 594 (29.1) | 665 (32.2) |
| Moderate | 514 (25.0) | 495 (24.1) | 492 (24.1) | 509 (24.7) |
| High | 884 (43.1) | 886 (43.2) | 955 (46.8) | 890 (43.1) |
| Smoking, n (%) |  |  |  |  |
| Current | 152 (7.8) | 155 (8.0) | 130 (6.7) | 111 (5.6) |
| Former | 923 (47.4) | 922 (47.4) | 955 (48.9) | 968 (49.2) |
| Never | 871 (44.8) | 868 (44.6) | 866 (44.4) | 888 (45.1) |
| BMI, kg/m2 | 25.6 ± 4.2 | 25.5 ± 4.3 | 25.4 ± 4.1 | 25.6 ± 4.2 |
| Weight status |  |  |  |  |
| Normal weight, n (%) | 987 (48.5) | 1040 (51.3) | 1035 (51.2) | 1009 (49.1) |
| Overweight, n (%) | 781 (38.3) | 735 (36.3) | 745 (36.9) | 774 (37.7) |
| Obesity, n (%) | 269 (13.2) | 251 (12.4) | 241 (11.9) | 272 (13.2) |
| Kcal/d | 1,901 ± 646 | 1,885 ± 639 | 1,904 ± 639 | 1,881 ± 634 |
| DHD-15 food groups, g/day |  |  |  |  |
| Vegetables | 120 (73 - 171) | 116 (67 - 172) | 121 (71 - 177) | 117 (69 - 172) |
| Fruit | 176 (81 - 237) | 163 (77 - 236) | 151 (71 - 234) | 154 (74 - 236) |
| Wholegrain bread | 70 (24 - 106) | 70 (18 - 106) | 70 (22 - 109) | 70 (23 - 106) |
| Legumes | 6 (0 - 17) | 6 (0 - 17) | 6 (0 - 17) | 6 (0 - 17) |
| Nuts | 6 (1 - 21) | 6 (1 - 19) | 7 (1 - 20) | 7 (1 - 20) |
| Dairy | 266 (142 - 399) | 262 (139 - 397) | 258 (143 - 403) | 263 (143 - 402) |
| Fish | 17 (7 - 36) | 14 (7 - 29) | 14 (7 - 29) | 14 (7 - 36) |
| Tea | 340 (121 - 510) | 340 (121 - 510) | 340 (121 - 680) | 340 (146 - 680) |
| Butter and solid fats | 0 (0 - 5) | 0 (0 - 6) | 0 (0 - 6) | 0 (0 - 6) |
| Oils and diet margarines | 11 (3 - 31) | 11 (3 - 31) | 11 (3 - 31) | 12 (3 - 30) |
| Red meat | 42 (19 - 75) | 40 (18 - 76) | 39 (17 - 74) | 40 (18 - 75) |
| Processed meat | 25 (10 - 44) | 23 (8 - 42) | 22 (9 - 42) | 23 (9 - 41) |
| Sweetened beverages and fruit juices | 68 (9 - 179) | 57 (6 - 175) | 64 (7 - 175) | 75 (9 - 182) |
| Alcohol | 9 (1 - 22) | 9 (0 - 22) | 10 (1 - 22) | 8 (1 - 20) |
| Neighbourhood socioeconomic status^b^ | 0.2 (-0.5; 0.9) | 0.2 (-0.7; 1.1) | 0.4 (-0.3; 1.1) | 0.4 (-0.4; 1.1) |
| Level of urbanization^c^, n (%) |  |  |  |  |
| Very low level of urbanisation | 506 (25.7) | 753 (37.0) | 706 (35.4) | 469 (23.0) |
| Low level or urbanisation | 607 (30.9) | 580 (28.5) | 648 (32.5) | 694 (34.0) |
| Moderate level of urbanisation | 375 (19.1) | 305 (15.0) | 318 (15.9) | 503 (24.6) |
| High level of urbanisation | 242 (12.3) | 256 (12.6) | 200 (10.0) | 156 (7.6) |
| Very high level or urbanisation | 236 (12.0) | 141 (6.9) | 124 (6.2) | 219 (10.7) |

^a^Continuous variables are presented as means (standard deviation) or as medians (p25 – p75). ^b^Higher scores represent higher neighbourhood socioeconomic status. ^c^Very low level of urbanisation ≤ 500 addresses/km^2^; low level of urbanisation = 500-1000 addresses/ km^2^; moderate level of urbanisation = 1000-1500 addresses/ km^2^; high level of urbanisation =1500-2000 addresses/ km^2^; very high level or urbanisation ≥ 2000 addresses/ km^2^. The following variables had missing data: smoking status (n=422); level of urbanisation (n=193); BMI (n=92); household educational level (n=22); neighbourhood socioeconomic status (n=16).
