# Supplementary material for: Residential exposure to fast-food restaurants and its association with diet quality, overweight and obesity in the Netherlands: a cross-sectional analysis in the EPIC-NL cohort
Source: Nutr J. 2021 Jun 16;20:56. doi: 10.1186/s12937-021-00713-5 (PMC8210363; doi:10.1186/s12937-021-00713-5)
Supplement: Supplementary file 4 — Additional file 4. Participant characteristics across quartiles of FFR proportion in the 1500m buffer. [file 12937_2021_713_MOESM4_ESM.docx]

**Additional file 4.** Participant characteristics across quartiles of FFR proportion in the 1500m buffer^a^.

|  | Q1 | Q2 | Q3 | Q4 |
| --- | --- | --- | --- | --- |
| N, (%) | 2057 (25.0) | 2060 (25.0) | 2128 (25.9) | 1986 (24.1) |
| Median FFR proportion | 0.10 (0.00 - 0.10) | 0.13 (0.12 - 0.13) | 0.17 (0.16 - 0.19) | 0.22 (0.20 - 0.24) |
| Age, y | 70 ± 10 | 71 ± 10 | 69 ± 10 | 70 ± 10 |
| Sex, n (%) |  |  |  |  |
| Male | 367 (17.8) | 445 (21.6) | 442 (20.8) | 365 (18.4) |
| Female | 1690 (82.2) | 1615 (78.4) | 1686 (79.2) | 1621 (81.6) |
| Household educational level, n (%) |  |  |  |  |
| Low | 639 (31.2) | 681 (33.1) | 666 (31.4) | 598 (30.2) |
| Moderate | 507 (24.7) | 481 (23.4) | 534 (25.2) | 488 (24.6) |
| High | 905 (44.1) | 895 (43.5) | 921 (43.4) | 894 (45.2) |
| Smoking, n (%) |  |  |  |  |
| Current | 158 (8.1) | 136 (7.0) | 149 (7.3) | 105 (5.6) |
| Former | 921 (47.0) | 948 (49.0) | 965 (47.6) | 934 (49.5) |
| Never | 879 (44.9) | 851 (44.0) | 915 (45.1) | 848 (44.9) |
| BMI, kg/m2 | 25.4 ± 4.3 | 25.7 ± 4.2 | 25.6 ± 4.3 | 25.5 ± 4.2 |
| Weight status |  |  |  |  |
| Normal weight, n (%) | 1042 (51.3) | 994 (49.0) | 1055 (50.0) | 980 (49.8) |
| Overweight, n (%) | 734 (36.1) | 780 (38.4) | 788 (37.3) | 733 (37.3) |
| Obesity, n (%) | 255 (12.6) | 256 (12.6) | 269 (12.7) | 253 (12.9) |
| Kcal/d | 1,876 ± 637 | 1,913 ± 654 | 1,897 ± 632 | 1,884 ± 634 |
| DHD-15 food groups, g/day |  |  |  |  |
| Vegetables | 119 (72 - 173) | 119 (66 - 173) | 118 (71 - 172) | 120 (71 - 173) |
| Fruit | 165 (77 - 236) | 163 (77 - 236) | 155 (71 - 236) | 161 (79 - 235) |
| Wholegrain bread | 70 (21 - 106) | 70 (18 - 106) | 70 (19 - 106) | 71 (29 - 106) |
| Legumes | 6 (0 - 17) | 6 (0 - 17) | 9 (0 - 17) | 6 (0 - 17) |
| Nuts | 6 (1 - 20) | 6 (1 - 20) | 6 (1 - 19) | 7 (2 - 21) |
| Dairy | 269 (138 - 400) | 258 (144 - 397) | 255 (142 - 401) | 268 (145 - 401) |
| Fish | 18 (7 - 36) | 14 (7 - 29) | 14 (5 - 32) | 14 (7 - 29) |
| Tea | 340 (121 - 510) | 340 (146 - 583) | 340 (109 - 510) | 340 (121 - 680) |
| Butter and solid fats | 0 (0 - 5) | 0 (0 - 7) | 0 (0 - 5) | 0 (0 - 6) |
| Oils and diet margarines | 11 (3 - 31) | 12 (3 - 32) | 11 (3 - 28) | 11 (3 - 32) |
| Red meat | 39 (17 - 73) | 41 (19 - 79) | 40 (18 - 75) | 40 (18 - 74) |
| Processed meat | 23 (9 - 43) | 24 (9 - 44) | 23 (9 - 43) | 23 (9 - 40) |
| Sweetened beverages and fruit juices | 71 (13 - 176) | 63 (6 - 175) | 66 (7 - 180) | 69 (9 - 175) |
| Alcohol | 9 (1 - 22) | 10 (0 - 22) | 9 (0 - 21) | 8 (1 - 20) |
| Neighbourhood socioeconomic status^b^ | 0.2 (-0.4; 0.9) | 0.2 (-1.0; 1.1) | 0.4 (-0.4; 1.0) | 0.5 (-0.2; 1.1) |
| Level of urbanization^c^, n (%) |  |  |  |  |
| Very low level of urbanisation | 566 (28.7) | 796 (39.1) | 674 (32.2) | 398 (20.5) |
| Low level or urbanisation | 620 (31.5) | 590 (29.0) | 635 (30.3) | 684 (35.3) |
| Moderate level of urbanisation | 311 (15.8) | 294 (14.5) | 411 (19.6) | 485 (25.0) |
| High level of urbanisation | 241 (12.2) | 220 (10.8) | 236 (11.3) | 157 (8.1) |
| Very high level or urbanisation | 232 (11.8) | 134 (6.6) | 140 (6.7) | 214 (11.0) |

^a^Continuous variables are presented as means (standard deviation) or as medians (p25 – p75). ^b^Higher scores represent higher neighbourhood socioeconomic status. ^c^Very low level of urbanisation ≤ 500 addresses/km^2^; low level of urbanisation = 500-1000 addresses/ km^2^; moderate level of urbanisation = 1000-1500 addresses/ km^2^; high level of urbanisation =1500-2000 addresses/ km^2^; very high level or urbanisation ≥ 2000 addresses/ km^2^. The following variables had missing data: smoking status (n=422); level of urbanisation (n=193); BMI (n=92); household educational level (n=22); neighbourhood socioeconomic status (n=16).
